# Use of *in vitro* fertilization—ethical issues

**DOI:** 10.1080/03009734.2019.1684405

**Published:** 2019-11-05

**Authors:** Kjell Asplund

**Affiliations:** Department of Public Health and Clinical Medicine, Umeå University, Umeå, Sweden

**Keywords:** Age limits, ethics, *in vitro* fertilization, infertility, preimplantatory genetic testing, public funding, prioritization

## Abstract

This report is an ethical analysis based on both facts and values. In *in vitro* fertilization (IVF), there is an intricate interaction between rapid scientific development and changing societal values. In most countries, the ethical discussion is no longer on whether or not IVF in itself is ethically justifiable. Therefore, in this review, I discuss other ethical aspects that have emerged since IVF was first introduced, such as upper age limits, ‘ownership’ of gametes and embryos, IVF in single women and same-sex couples, preimplantatory genetic testing, social egg freezing, commercialization, public funding, and prioritization of IVF. Despite secularization, since religion still plays an important role in regulation and practices of IVF in many countries, positions on IVF among the world religions are summarized. Decision-making concerning IVF cannot be based only on clinical and economic considerations; these cannot be disentangled from ethical principles. Many concerns regarding the costs, effects, and safety of IVF subtly transcend into more complex questions about what it means to society to bear and give birth to children.

## Introduction

Ethics is about the systematic reflection on human values and actions. Simplistically, the two principal components of an ethical analysis are facts and values. When facts and values change, the ethical analysis must be revised.

*In vitro* fertilization (IVF) is an illustrative case of this dynamic process. In some aspects, the expansion of scientific information has made decision-making easier; it has left less room for opinions and speculations, and facilitated evidence-based regulations and clinical recommendations. On the other hand, rapid technological development has implied a number of new ethical challenges.

Since medical ethics has a history that goes back to the Hippocratic principles, it would be easy to conclude that values are, or should be, unwavering. It is then thought-provoking to view IVF as an example of how radically societal values and attitudes have changed over the last four decades.

Given the changes in facts and values, the prerequisites for an ethical analysis of IVF are under ongoing evolution. In most countries, the ethical discussion is no longer on whether or not IVF in itself is ethically justifiable. Therefore, the major focus of this article is on new ethical questions that are invoked by biotechnological development or changes in societal values.

The focus of this article is on IVF practices and ethical discussions in Europe and English-speaking countries. This is where most of the scientific literature on ethical issues on IVF originates, but it inevitably means that this article is ethnocentric. Several examples of ethical analyses are taken from my work as Chair of the Swedish Council on Medical Ethics, which advises the Swedish Government and Parliament on medical ethics issues.

I will discuss ethical aspects on upper age limits, ‘ownership’ of stored gametes and embryos, IVF in single women and same-sex couples, preimplantatory genetic diagnosis, social egg freezing, egg sharing, surrogacy, commercialization, public funding, and prioritization of IVF. Finally, despite secularization, since religion still plays an important role in the regulation and practices of IVF in many countries, positions on IVF among the world religions are summarized.

## Brief historical notes

The ethical controversy over IVF had already started when the results of the first animal IVF experiments were published in the mid-1930s (the results were later contested). While some commentators saw it as a promising development to help infertile couples, others were critical, saying that the scientists were playing God (1). It is interesting to note that the animal experiments evoked eugenic questions that were in focus at the time. In a 1936 article in *New York Times*, it was stated: ‘Advocates of “race betterment” might urge such procedures for men and women of special aptitudes, physical, mental, or spiritual’ (cited by Biggers (1)). In the press, there was speculation, repulsive in nature, on both surrogacy and a world where men were not needed for reproduction: ‘The mythical land of the Amazons would then come to life’ (1).

In 1978, when the first child was born after IVF in the UK, the negative ethical comments of the 1930s were reiterated with more fervour. The protests seem to have been particularly strong when attempts were made to introduce IVF in the USA. John Biggers, one of the IVF pioneers, recalled memories from a public hearing arranged by the state of Virginia: ‘The entire meeting turned out to be a shouting match, the two groups [“pro-lifers” and IVF proponents] hurling insults at each other’ (1).

In the 40 years since the first successful human IVF, enormous experience and scientific knowledge have accumulated. Few of the fears of negative effects have been met. Values have changed, partly because more facts are available, but also due to general trends in values in many populations, secularization perhaps being the most important. Today, the principal resistance to IVF as such rests mainly in Catholic and Orthodox Christian settings. The philosophical argument that being born is better than not being born has also been invoked in the IVF debate (the opposing view is abundantly present in philosophy as well as religion, for example elaborated by the philosopher David Benatar in his book *Better Never to Have Been: The Harm of Coming into Existence* (2)).

## Age limits

Upper-middle-age women who give birth to a child after IVF are often subject to public opinion on how ‘unfitting’, ‘unnatural’, and even ‘repulsive’ this is. Table 1 summarizes the principal arguments for and against a fixed upper age limit for IVF in older women. In the public debate, the age limit discussed is usually in the 40–50-year age range.

**Table 1.** Four common arguments supporting and four arguments against an upper maternal age limit for IVF.

Supporting age limit	Against age limit
A child has a right to a safe childhood and adolescence. This right may be encroached upon when the mother has an age-related risk of disease and possibly death. The woman and the foetus are at increased risk with advancing maternal age. With advancing maternal age, the chance of successful IVF decreases; if there is public financing of IVF, this argument includes cost-effectiveness and prioritization deliberations. If donated eggs are used, limited availability of eggs may justify that younger women with strictly ‘medical’ infertility and a higher chance of successful pregnancy are prioritized over women with age-related infertility.	Reproductive autonomy: a woman should have the right to choose when she wants to have a child. With secular trends of improved physical health in upper middle age, the prerequisites for being a healthy and active mother have improved. Chronological age has therefore become less decisive when parenthood is considered. Other factors, such as the presence of chronic life-threatening disease or severe alcohol and drug abuse, are more crucial. Middle-aged women are often psychologically mature and have a stable social and economic situation; the preconditions for care of a child are usually good. An upper age limit means formalized age discrimination.

Thus, the arguments concern both facts and values. The likelihood of outcomes such as successful pregnancy or the death of the mother before the child is able to support itself are examples of fact-based arguments, as are the consequences of the outcomes. The most obvious value-based arguments are the right of children to be brought up under safe conditions and women’s reproductive autonomy.

Some arguments are based on both facts and values. Are older women as good mothers as younger women? The answer may be value-based, but it may also be based on the scientific information available. There have been reports that children born to mothers above the age of 35 or 40 do not have increased physical or mental vulnerability, but a modest increase in vulnerability has also been reported at maternal ages above 35 (3). Yet, at maternal ages above 35 or 40 years, child vulnerability still seems to be consistently lower than that in children born to very young mothers (3).

Compared to upper age limits for the mother, there has been little discussion on age limits for the father. Men are, on average, older than women when they become parents and have a somewhat shorter life expectancy than women. Therefore, the argument of being able to take responsibility for a child until adulthood would apply equally, if not more, to men as to women.

After considering basic ethical principles and the large variations in individual prerequisites, both medically and socially, the Swedish Council on Medical Ethics has recommended that there should be no strict upper age limit for publicly funded IVF (4). Instead, individual assessments should be made, taking into consideration biological rather than chronological age. The needs of the child are emphasized: at least one parent should be young enough to take responsibility for the child until adulthood.

When political decisions are made, other ethical values and non-ethical factors may play a major role. In 2016, public healthcare providers in Sweden agreed to apply a national upper age limit for publicly funded IVF (5). The age limit was set at 40 years in women and 56 years in men. Stored embryos could be used up to a maternal age of 45. A major reason for this decision was to eliminate the then-existing regional variations in age limits. The principles of fairness, cost-effectiveness, and simplicity in decision-making seem to have overridden other ethical principles, most importantly reproductive autonomy and non-discrimination by age. In Swedish private practice with out-of-pocket payments, more variable age limits are applied, usually well above 40 years.

In most countries, there are no national age limits for IVF, and practices vary. In the UK, for instance, public funding is decided locally by Clinical Commissioning Groups (CCGs), with different practices across the country. There are, however, healthcare professionals’ guidelines that recommend that women up to the age of 40 should be offered three cycles of IVF and women up to the age of 42 one cycle of IVF (6).

## Single women and same-sex couples

The major ethical questions about providing IVF or other forms of assisted reproduction for single women and same-sex couples have concerned the welfare of the child. Ethical arguments for and against IVF in singles and homosexuals that have been discussed are those of fairness, non-discrimination, reproductive autonomy, and children’s well-being.

A systematic literature review (with methodological problems in most of the included studies) compared adolescents born through IVF for single women or women in same-sex partnerships with naturally conceived adolescents. There were no differences in psychological adjustment or parent–adolescent relationships (7).

Not surprisingly, legal restrictions on IVF and other forms of assisted reproduction for singles and same-sex couples vary between countries. In a survey of legislation in the 28 EU countries published in 2014, medically assisted reproduction (including IVF) for single women was permitted in 11 countries and not allowed in 11 countries, whereas the legal status was undefined in the remaining 6 countries (8). Since 2014, some countries with a previous ban have modified the legislation to permit IVF for single women. It is difficult to discern a distinct pattern of countries permitting or prohibiting IVF as to traditional values, level of secularity, or extent of self-expression in the population [see World Values Survey (9)].

Some regulatory differences exist even between countries where assisted reproduction is permitted for single women. In Sweden, a child born through gamete donation has the right to know how it was conceived and who is the genetic father and mother; this is based on the principle of autonomy. Some Swedish women prefer to have IVF performed in Denmark, where the anonymity of the sperm donor is ensured.

For female same-sex couples, there is usually no specific legislation; when IVF for single women is allowed, this would also cover female same-sex couples. In a 2013 statement by its ethics committee, the American Society for Reproductive Medicine called for programmes providing IVF and other fertility services to ‘treat all requests for assisted reproduction equally without regard to marital/partner status or sexual orientation’ (10). In most countries where surrogacy is allowed (or not prohibited by law), IVF with sperm from a homosexual father-to-be may be used for conception of the surrogate mother.

## Who ‘owns’ stored gametes and embryos?

Humans have always strived for a form of immortality through our genetic offspring (11). Gamete storage has now enabled us to plan prospectively, allowing for untimely demise or social reproductive choices. The aim is that our genetic ‘identity’ should continue after our death. Aside from what the deceased person may have wanted, it is often a wish of the family to immortalize their loved one. For the family, use of stored gametes (or embryos) may be a relief from part of the sorrow (11).

Thus, when gametes are collected and stored before cancer treatment and the patient dies, should the remaining partner have a right to use them for IVF? In practice, this issue occurs when the remaining partner is a woman, but hypothetically a remaining male partner could request to use an egg from a deceased female partner to engage a surrogate mother.

Would a person who stores sperm, eggs, or embryos choose to reproduce after death? Anyone who has decided to store them for future reproduction could perhaps be presumed to have consented. Usually, however, the consent does not involve the event that the person dies. Use of gametes from deceased individuals is currently prohibited by law in France, Germany, Sweden, and other countries, even when there is written consent from the deceased. In the UK, sperm from a deceased person can be used for IVF if there is written consent before death. In the USA, practices vary from clinic to clinic, and written consent is not always required (12).

Postmortem sperm retrieval (PMSR) raises significant ethical and legal concerns, including issues of implied/presumed consent and the designation of sperm as property. In many countries, the legal situation is not clear. Most commentators seem to agree that a core principle is not to reproduce anyone without his or her permission (12). Thus, consent should be obtained. In countries where the use of gametes from deceased individuals is prohibited (see above), any use of PMSR would require a modification of the legislation.

Grieving family members are not always in the position to make rational choices. Therefore, some experts have recommended compulsory wait times (up to one year) before using the retrieved sperm for conception.

If a couple who have decided to store embryos for future use splits up and only one of the partners wants to use them (in a hypothetical case, the male partner may want to use the embryos for surrogacy), who ‘owns’ the embryos and has the right to decide (it would be more appropriate to talk about disposal than strict ownership)?

A UK case attracting much public attention may illustrate the ethical and legal complexity. Embryos were stored before oophorectomy in a woman with ovarian cancer (13). When the couple split up half a year later, the man wanted the embryos to be destroyed. The clinic informed the woman that UK laws requested consent from both partners for storage to continue. The woman brought the case to court, all the way to the Grand Chamber of the European Court of Human Rights. The courts, both British and European, decided against the woman’s wishes (but the decisions were not always unanimous). The legal—and ethical—dilemma was: how to weigh the need for consent from both partners against the right to a family life as enshrined in the European Convention of Human Rights. The Grand Chamber decided that the right to a family life could not override the male partner’s withdrawal of consent (13). Thus, the principle of consent has strong legal support of the European Court.

## IVF and preimplantatory genetic testing (PGT)

The fact that IVF may be used for purposes other than the treatment of infertility evokes additional ethical questions and dilemmas.

In families in which a child with a severe monogenetic disease has previously been born or if there is a high risk of aneuploidy, preimplantation genetic testing (PGT; previously PGD, preimplantation genetic diagnosis) offers a way to escape a pregnancy with a severely diseased child. A possible late-term abortion or the birth and early death of an infant may be avoided. For early-onset severe or lethal monogenetic diseases and structural chromosome rearrangements, the use of PGT is relatively non-controversial ethically.

Other applications of PGT have raised ethical concerns. The most obvious question is: What diseases and aneuploidies should be assessed? Increasingly, PGT is being considered for late-onset, variably penetrant, and less severe conditions.

Even more ethically challenging: could testing be based on non-disease characteristics with genetic influences, such as intelligence and beauty (what has been called ‘preimplantatory genetic profiling’)? The concern that such testing would lead to choosing a child to order, as a commodity that has been designed simply to meet the needs and desires of the parents (‘catalogue babies’), was raised already when PGT was introduced in the 1990s (14). This prospect has created fears that the increasing frequency of ‘genetic profiling’ will move toward a modern eugenics movement (15).

PGT can also be used to select embryos by sex and thus reach what has been referred to as ‘family balancing’. Particular concern has arisen when IVF has been used for selection by sex in populations where male offspring are favoured over female offspring for cultural and economic reasons. In the UK, the use of PGT for sex selection of embryos for non-medical reasons is explicitly forbidden (16).

Highly publicized cases of ‘savior siblings’ have concerned the use of preimplantation tissue typing (PTT) to select embryos that could produce children suitable to become donors of stem cells or tissues for siblings who suffer from severe diseases (17). The merits of saving a sibling can be rationalized and commended. However, in other organ and tissue donation, treatment of a person solely for the purpose of becoming a donor is rejected. In PTT, questions on consent and the protection of children’s autonomy become paramount.

The regulation of PGT and PTT varies between countries, often also between regions in the same country. In most EU countries, PGT is allowed, either by legislation or because there is no explicit prohibition (18). The most detailed regulation is probably in the UK, where the Human Fertilization and Embryology Authority (HFEA) lists more than 600 genetic conditions for which PGT is allowed. These conditions have also been approved for use in cases involving PTT. However, unlike PGT, PTT requires additional approval on a case-by-case basis for specific patients (19).

There are attempts to harmonize the use of PGT in EU countries. The European Court of Human Rights has sanctioned Italy and Latvia for refusing access to PGT. The Court refers to the right to bring a child into the world who is not affected by the illness that they carry (20, 21). In other countries, such as Canada and the USA, there is no national regulation of PGT. It has been argued that not regulating PGT also involves taking a moral position (22).

## Storage of oocytes for social reasons

Since the success rate of IVF declines rapidly in ages above 35 years when the woman uses her own eggs, social egg freezing (oocyte cryopreservation) has been introduced as a means to preserve and store oocytes retrieved at an earlier age. Stored oocytes are used in IVF at a time when the social circumstances for having a child would be preferable (23).

Ethical considerations that have evolved in the discussions on social oocyte cryopreservation have concerned reproductive autonomy, risks involved in egg retrieval, undue hope, risk of failure, and the need for truly informed consent. To this end, the pros and cons of an upper age limit for IVF have been added (see above).

Most countries do not have national regulations on social oocyte cryopreservation. One exception is Israel, where the procedure has been regulated and authorized for public support. The main justification has been that of promoting individual autonomy (24). When the Swedish Council on Medical Ethics reviewed social egg freezing, the medical problems with delayed motherhood were weighed against reproductive autonomy. The Council found no convincing ethical arguments for a ban on social egg freezing, but concluded that the costs should not be covered by public funding (4).

## Egg sharing

The term *egg sharing* is used when a woman who is already having IVF donates some of her eggs to other women. This may be done as an entirely altruistic act, but the eggs may also be donated to the clinic where she is having treatment in return for free or discounted treatment. If so, an indirect form of economic incentive is introduced. This is accepted in some countries, whereas other countries have taken a universal position against commercialization of organ or tissue donation, including donation of gametes; only modest reimbursement for expenses is allowed.

In the UK, where egg sharing in return for free treatment is allowed, The Human Fertilization and Embryology Authority emphasizes that the woman should never be put under any pressure to share her eggs. She should be informed that egg sharing is a big decision with serious implications, and she should receive professional counselling before going ahead. In the UK, as in some other countries, this includes information about the right of children conceived by egg sharing to know about their genetic origin when they turn 18 (25).

## Surrogacy

The term *surrogacy* has in itself a judgmental connotation (and the term *surrogate children* is even more objectionable). But since the terminology battle now seems to be over (even among healthcare professionals), I am, somewhat reluctantly, using *surrogacy* here.

Surrogacy may either be partial, when pregnancy is initiated through insemination, or full, involving eggs from another woman than the surrogate mother. In full surrogacy, eggs and sperm may come from the intended parents or from external donors. Full surrogacy thus involves IVF with no genetic link to the surrogate mother but with genetic link(s) to two, one, or none of the intended parents.

The ethics of surrogacy, whether partial or full, is covered by an abundance of analyses and debate articles (26). Much of the ethics debate is on altruistic versus commercial surrogacy, autonomy versus exploitation of women, human dignity, medical risks, balancing interests of the persons involved and the long-term well-being of surrogate mothers, children, and their families.

However, the ethical debate has only rarely distinguished between partial and full surrogacy. Most intended parents seem to prefer to maximize the genetic linkage to the extent that is medically feasible. IVF then becomes the method of choice for conception, and this may be regarded as catering for the principle of autonomy. An additional possible argument for full surrogacy is that the psychological impact would conceivably be less if the surrogate mother knows that she is not genetically related to the child she is carrying and will be separated from.

## Commercialization of IVF

With the increasing demand for IVF, the economic impact of the IVF sector has come into focus highlighting the possible negative aspects of the commercialization of IVF. Ethical questions that are often raised in the debate include equity, possible exploitation of need and hope, consent that is truly informed, and the many components of marketing ethics.

The ‘IVF industry’ has been seen as an example of what social scientists describe as an increasing trend toward a market-driven construction of health, medicine, and the human body (27). Most of the public debate on commercialization of IVF has not, however, concerned IVF as such but the reimbursement of gamete donors (egg donors in particular), the selling of embryos, and the use of IVF for commercial surrogacy.

## Public funding of IVF and prioritization

Whether or not IVF should be funded publicly is, to a large extent, a matter of priority ethics. The Swedish model for priority-setting in healthcare may serve as an example. It is based on three ethical principles (28).

### The human dignity principle

This is basically a principle of equal value, equal human rights, and non-discrimination. All people have human dignity simply by being human and not for what they have or do. Access to healthcare services should not depend on sex, social or economic background, religion, sexual orientation, or cognitive function, among others.

According to this principle, singles and homosexuals should have the same right to IVF as infertile heterosexual couples in need of sperm or egg donation. Regional and socioeconomic differences in access to public funding of IVF are not in accordance with the principle of human dignity. It has been argued that the right to health, as defined by the World Health Organization, includes the right to have children. Even if this perspective is accepted, it does not necessarily translate into a right to public funding of IVF (29).

### The needs and solidarity principle

Resources should be allocated to patients who have the greatest need. Need is assessed by (a) the severity of the health problem; (b) the potential health improvement that would be brought about by a healthcare intervention; and (c) the scientific support for a favourable benefit–risk ratio. The solidarity component means that special attention should be paid to those who cannot themselves express their needs, such as children, people with dementia, or patients with severe mental disorders. The principle of needs and solidarity contrasts with requests for healthcare, often not equalling needs.

The main ethical debate in the treatment of infertility concerns need. There are many individual variations in how involuntary childlessness is perceived, and these may be affected by societal norms, attitudes of next-of-kin and friends, how childlessness is presented in the media, and other factors. Decisions on public funding of IVF are, however, not made at the individual level but at group levels.

### The cost-effectiveness principle

According to this principle, the healthcare system has a duty to utilize its resources as effectively as possible, and there should be a reasonable balance between the costs and effects of an intervention. This principle is subordinate to the other two principles.

The cost-effectiveness of IVF primarily depends on three factors: (a) treatment success rates; (b) multiple pregnancies; and (c) the cost of treatment (29). The cost-effectiveness has been estimated to be notably lower in older than in younger women (30), so the (subordinate) cost-effectiveness principle may conflict with the human dignity principle. A weakness in the cost-effectiveness studies is that costs are usually measured per successful outcome such as live births or ‘take-home babies’. In other medical areas, costs are usually expressed as costs per quality-adjusted (QALYs) or disability-adjusted life years (DALYs) gained. The different denominators make it difficult to prioritize IVF versus other healthcare interventions (horizontal priority-setting).

The three principles for priority-setting provide an ethical platform, but they do not resolve all issues regarding the prioritization of IVF. A frequent question is: Should women and men with an established medical cause of infertility have a higher priority for assisted reproduction than those with unexplained infertility? Since the medical preconditions may differ, the need can, at least to some extent, be expressed in term of effects, adverse effects, and the strength of scientific documentation. But usually, needs in terms of distress and suffering do not differ by cause of infertility; the existential burden remains the same. Therefore, it would not be acceptable to prioritize IVF solely based on the presence or absence of an established medical cause of infertility. It has also been argued that women with age-related infertility have a greater need for IVF since they are more likely to be permanently infertile and their time to become pregnant is running out.

Public funding of IVF varies between countries. In North America, payment for IVF is usually through private health insurance or out-of-pocket funds. In the USA, high costs have generated ‘reproductive tourism’ to countries with lower fees but also with safety concerns (31). In many European countries, there are programmes for public funding but with considerable variations in the number of cycles that are funded, age limits, and the proportion of the total cost that is paid for, among other factors (29,32). Public funding is often restricted to IVF performed in public hospitals and clinics (29).

In public funding, equity is an important aspect of the human dignity principle. It has repeatedly been shown that socioeconomically disadvantaged groups have less access to assisted reproduction services than more privileged groups. Socioeconomic disparities persist after adjusting for several confounding factors, including age at first birth and geographic remoteness (33). In Finland, where IVF is publicly funded whether performed in public or private clinics, socioeconomic inequities have been observed in private but not public clinics (34).

## IVF and religion

While academic bioethics usually considers both facts and values when performing ethical analyses, religions rely principally on values. Religious beliefs may be decisive when infertile women and men consider the IVF option. They may also impact law-making and other regulations in a country.

IVF and procedures associated with IVF are summarized by religion in Table 2. By necessity, the table gives a very simplified view. Within one religion, different sects have diverse interpretations and have reached varying conclusions. Some of the information in the table is from unofficial Internet sources; the summary should therefore be regarded as provisional. The text below comments on the religions’ positions on IVF.

**Table 2.** IVF by religion and cultural tradition.

Religion/cultural tradition	IVF	IVF for singles	Gamete donation	Embryo donation	Surrogacy through IVF
Christianity					
Catholic	No	No	No	No	No
Orthodox	Yes/No	No	No	No	No
Protestant	Yes	Yes/No	Yes/No	Yes/No	No
Judaism	Yes	Debating	Yes	Yes/No	Yes/No
Islam					
Sunni	Yes	No	No	Debating	Yes
Shi’a	Yes	No	Yes	Yes	Yes
Hinduism	Yes	No	Sperm only, conditional	Yes	Yes
Buddhism	Yes	No	Yes	Yes	Yes
China^a^	Yes	Debating	No	No	No
Japan^a^	Yes	No	Sperm only	No	No

^a^ Emerging from legislation. Modified and expanded from Sallam and Sallam (36). See the text for additional restrictions and other comments.

The Roman Catholic Church opposes IVF. In 2007, Pope Benedict XVI declared that IVF and other forms of assisted reproduction are unworthy methods of conception, since they separate the procreative goal of marital sex from the goal of uniting a married couple. An additional reason for the resistance against IVF is that some embryos (beginnings of a new lives) are discarded (35).

The position of the Eastern Orthodox Churches on IVF seems to be somewhat less restrictive than that of the Catholic Church. Under some circumstances, the Eastern Orthodox Church permits the use of parents’ gametes for IVF, fertilising only as many embryos as will be implanted, thus avoiding scenarios under which embryos are discarded (36).

Among the Protestant Churches, there is no common statement on IVF. In most Protestant countries, IVF as such is no longer disputed, but some of the applications are questioned. For instance, the Church of England has expressed profound concern at offering fertility treatments to single women and gay couples (36).

Followers of the Jewish faith are encouraged to have children (‘Be fruitful and multiply, fill the earth and subdue it’, Genesis 1:28). IVF is allowed, and in Israel it is encouraged. Certain aspects of assisted reproduction are still controversial among Orthodox Jews, for example the collection of sperm (the ‘spilling of seed’ is prohibited) and donation of gametes and embryos (36).

Islamic jurisprudence states that any act is permissible unless prohibited by a text in the Quran. Islam affirms the importance of marriage, family formation, and procreation. According to Sunni Islam fatwas (religious opinions/rulings), all forms of assisted reproduction are allowed as long as the sperm and oocyte are those of the husband and his wife (37). A third party cannot be involved in the conception; that is, gamete or embryo donation is not allowed. Most Sunni Muslims accept surrogacy, provided that the gametes come from the prospective parents (37).

Shi’a principles and practices are similar to those in the Sunni fatwas, except that Shi’as permit gamete donation, the rationale being that it does not involve sexual intercourse with someone outside the family. Gestational surrogacy using IVF is accepted by Shi’a Muslims (36).

Hinduism is liberal with most assisted reproduction procedures, but demands that the egg and sperm come from a married couple. There are, however, exceptions: the sperm may also come from a close relative of an infertile man (36).

Buddhism is very permissive regarding IVF. It does not restrict the use of IVF to married couples, and sperm donation is permitted.

In Chinese and Japanese societies, there are influences from several religions, such as Buddhism, Confucianism, Taoism, and Shintoism. In addition, Japan is a highly secularized country, and in China it is common that several beliefs are practiced at the same time (it has been suggested that this should not be called ‘religion’ but rather ‘cultural practices’, ‘thought systems’, or ‘philosophies’ (36)). It is thus difficult to estimate to what extent religious beliefs have influenced the regulation of IVF and other forms of assisted reproduction in these countries. In China, IVF as such is permitted, but sex selection without medical indication, surrogacy, and gamete and embryo donation are prohibited (36). Japanese law permits IVF and sperm donation but not oocyte donation and surrogacy (36). After several landmark rulings by Japan’s Supreme Court, the Japan Society of Obstetrics and Gynecology has allowed unmarried couples access to IVF. Permission does not include single women or same-sex couples.

## Concluding remarks

This review has focussed on the individual woman and her partner. It should be added that, in some countries, IVF is used as a part of national policy to increase birth rates. South Korea (38) and Israel (39) have been exemplified as such pronatalist countries. If a pronatalist policy involves IVF promotion combined with a ban on abortions, as in South Korea, women’s reproductive health and rights are at stake (38).

This review has also aimed at showing that decision-making concerning IVF cannot be based only on clinical and economic considerations; they cannot be disentangled from ethical principles, nor from social, political, and philosophical considerations. As noted by Mladovsky and Sorenson (29), many concerns regarding the costs, effects, and safety of IVF subtly raise more complex questions about what it means to society to bear children.
